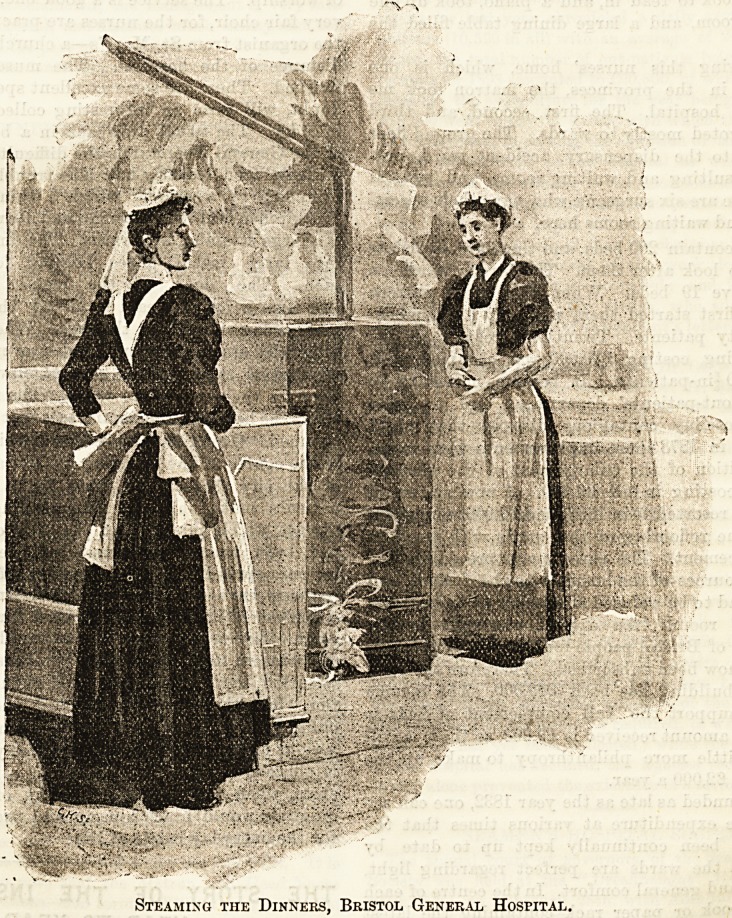# Bristol General Hospital

**Published:** 1894-03-10

**Authors:** 


### THE INSTITUTIONAL WORKSHOP.

### BRISTOL GENERAL HOSPITAL.

(By a Vagrant Correspondent.)

Between the River Avon and the floating harbour stands the Bristol General Hospital. Its left flank faces a piece of water called the Bathurst Basin. Approaching the building from the bridge spanning the lock, which connects the basin with the river, the hospital looks a very imposing pile, built of warm grey Bath stone in the modern Italian style, with lofty octagonal tower and cupola at one angle of the building. It looks like the superior hotel of a famous watering-place than an Hotel Dieu. Skirting the basin along its extensive flank one looks in vain for an entrance; in fact, I had to ask my way to the main doorway, and this I discovered round the corner, and then after much difficulty, for it was hemmed in on either side with very slummy types of houses; two of these were old time hostelries. One took the nautical name of the "Two Anchors," wine and spirit vaults; and the other the non-optional title of the "One Tap," with the notice that all beers were from the wood. The gateway of the hospital had one of these pubs for an immediate neighbour, and but for the imposing iron gate the visitor might have come to the conclusion that the landlord of the "One Tap" beer-house also ran a livery and bait stables; and this was the entrance to the mews. A very mewsy entrance it was, and as gloomy as a walled-in access to a prison.

Steaming the Dinners, Bristol General Hospital.

And still more gloomier was the courtyard into which this narrow passage led. It is difficult to describe this entrance to the Bristol General Hospital other than in a paradoxical manner. It is a "front-back" entrance, and a very poor entrance at that. The gloom of the approach to this hospital is soon dispelled on entering the building, for with the exception of the out-patients' quarters, which are on the ground floor and rather dark, the rest of the hospital is bright and cheery enough. The matron first of all took me to the new wing of the institute for nurses, which was admirable in every way. It is called the Edinburgh Wing, after the Duke of Edinburgh, who opened it 1891.

There was room here for forty private nurses, each nurse having her own comfortable bed-room. I went into several of these rooms and found them fitted much after the style of the students at Girton. There were no superfluous luggage allowed in these rooms. Trunks and heavy gear were left in a store-room below. The nurses' sitting-room was a spacious place, rather quaintly shaped, it being considerably narrower at one end, but it was charmingly furnished. A library, and a cosy nook to read in, and a piano, took up one side of the room, and a large dining table filled the other.

After viewing this nurses' home, which is one of the best in the provinces, the matron took me through the hospital. The first, second, and third floors are devoted mostly to wards. The ground floor is divided into the dispensary, accident ward, out-patients' consulting and waiting rooms, and general offices. There are six surgeons, who have their special consulting and waiting rooms here.

The wards contain 200 beds, and there are 45 sisters and nurses to look after them. The larger wards on each floor have 19 beds. When this great western hospital was first started there was only accommodation for thirty patients. Twenty-six years later the present building, costing £30,000, was occupied, with room for 150 in-patients. It became necessary to enlarge the out-patients' department and to build wards for possibly contagious diseases that might break out, so in 1873 these improvements were made, with the addition of the enlargement of the museum and library, costing in all £9,300. A new system of drainage was resorted to in 1883, and £9,000 were spent in this and the reflooring of the wards with wood in place of the cement. The three years preceding 1890 taxed the resources of the hospital to the fullest, and 1,517 cases had to be refused admission as in-patients for want of room. An appeal was made to the philanthropy of Bristol people for £20,000. The whole amount has now been subscribed. Therefore the total cost of the building has been £65,000. The income required to support the full complement of beds is £11,000; the amount received is £9,000, so there is still room for a little more philanthropy to make up the deficiency of £2,000 a year.

Though founded as late as the year 1832, one can see by the above expenditure at various times that the hospital has been continually kept up to date by reforms, and the wards are perfect regarding light, ventilation, and general comfort. In the centre of each ward is a book or paper rack containing the latest news and light literature for the use of the patients. Cheerful pictures decorate the walls, and ferns and flowers the tables and mantels. An extra blanket, red in colour, tucked in on one side of the beds, brighten up the ward. By-the-bye, the inventor and manufacturer of this comfort of civilisation was a Bristol man, Mr. Thomas Blanket, who in 1340, with several other inhabitants, set up looms in their homes for weaving woollen cloths, called after the inventor's name, blanket. At the end of each ward is a spacious sitting-room for the sisters. There are several small wards for special cases, and an excellent theatre and operating room, which is connected by an elevator to the several floors; the upper or third floor is occupied by the bed-rooms of the nurses and general staff.

The nurses' dining-room is large and bright, and occupies a part of the octagonal tower, and looks much like the ward-room of a battle ship. The sisters dine at one table, the nurses at another, and the private nurses dine together at a third. Unlike the Royal Infirmary on the other side of the town there is no special chapel; a large room in one of the angles of the building is arranged and used for a place of worship. The service is a good one, and there is a very fair choir, for the nurses are practiced in this by the organist from St. Mary's—a church within a short distance of the hospital. The museum is yet unfinished. There are some excellent specimens, and, no doubt, will form an interesting collection when once in order. The usual drawback in a building the size of the General Hospital is the difficulty in serving the patients' food decently hot. The trouble is successfully overcome by a system which I think is admirable. The kitchen is in the basement, and near the range there is a large steaming apparatus. The dinners are placed into large metal boxes holding forty-two plates each. The door of the metal box is closed, and then a jet of steam is forced through a pipe perforating the side of the case. When the boxes are filled with steam, they are handed over to the nurses, who wheel them to the general elevator and takes them to their respective wards. The matron superintends the heating process, and sees that each box is fully steamed. Owing to the position of this hospital being in the centre of the shipping quarter and busiest part of the city there is no space for a garden, so the patients make shift by promenading the spacious terrace which juts out at the back of the premises, and built over store houses and cellars. As this terrace spreads in the direction of the river the patients have a chance of a little fresh air; but a convalescent home is sadly wanted for this large hospital. The management has a supply of tickets for the western sanatorium, and two beds in another institution, but there is not half enough accommodation for the convalescent, and the majority are obliged to leave early to make room for the more serious cases. The result is that many a bread-winner is obliged to return to his labour still in a delicate state, when probably a few weeks of fresh air and quietude would brace him up and prepare him fully for the everyday battle of life.

## Figures and Tables

**Figure f1:**